# Platelet studies in autism spectrum disorder patients and first-degree relatives

**DOI:** 10.1186/s13229-015-0051-y

**Published:** 2015-10-23

**Authors:** Nora Bijl, Chantal Thys, Christine Wittevrongel, Wouter De la Marche, Koenraad Devriendt, Hilde Peeters, Chris Van Geet, Kathleen Freson

**Affiliations:** Department of Cardiovascular Sciences, Centre for Molecular and Vascular Biology, KU Leuven, Gasthuisberg Campus, O & N I, Herestraat 49-b911, 3000 Leuven, Belgium; Department of Neurosciences, Research Group Psychiatry, KU Leuven, Leuven, Belgium; Department of Human Genetics, KU Leuven, Leuven, Belgium; The LAuRes Consortium, KU Leuven, Leuven, Belgium

**Keywords:** Autism, Platelets, Dense granules, Platelet activation, ATP secretion, Serotonin

## Abstract

**Background:**

Platelets have been proven to be a useful cellular model to study some neuropathologies, due to the overlapping biological features between neurons and platelets as granule secreting cells. Altered platelet dense granule morphology was previously reported in three autism spectrum disorder (ASD) patients with chromosomal translocations that disrupted ASD candidate genes NBEA, SCAMP5, and AMYSIN, but a systematic analysis of platelet function in ASD is lacking in contrast to numerous reports of elevated serotonin levels in platelets and blood as potential biomarker for ASD*.*

**Methods:**

We explored platelet count, size, epinephrine-induced activation, and dense granule ATP secretion in a cohort of 159 ASD patients, their 289 first-degree relatives (103 unaffected siblings, 99 mothers, and 87 fathers), 45 adult controls, and 65 pediatric controls. For each of the responses separately, a linear mixed model with gender as a covariate was used to compare the level between groups. We next investigated the correlation between platelet function outcomes and severity of impairments in social behavior (social responsiveness score (SRS)).

**Results:**

The average platelet count was increased in ASD patients and siblings vs. controls (ASD 320.3 × 10^9^/L, *p* = 0.003; siblings 332.0 × 10^9^/L, *p* < 0.001; controls 283.0 × 10^9^/L). The maximal platelet secretion-dependent aggregation response to epinephrine was not significantly lower for ASD patients. However, secondary wave responses following stimulation with epinephrine were more frequently delayed or absent compared to controls (ASD 52 %, siblings 45 %, parents 53 %, controls 22 %, *p* = 0.002). In addition, stimulated release of ATP from dense granules was reduced in ASD patients, siblings, and parents vs. controls following activation of platelets with either collagen (ASD 1.54 μM, *p* = 0.001; siblings 1.51 μM, *p* < 0.001; parents 1.67 μM, *p* = 0.021; controls 2.03 μM) or ADP (ASD 0.96 μM, *p* = 0.003; siblings 1.00 μM, *p* = 0.012; parents 1.17 μM, *p* = 0.21; controls 1.40 μM). Plasma serotonin levels were increased for ASD patients (*n* = 20, *p* = 0.005) and siblings (*n* = 20, *p* = 0.0001) vs. controls (*n* = 16). No significant correlations were found in the different groups between SRS scores and count, size, epinephrine aggregation, or ATP release.

**Conclusions:**

We report increased platelet counts, decreased platelet ATP dense granule secretion, and increased serotonin plasma levels not only in ASD patients but also in their first-degree relatives. This suggests that potential genetic factors associated with platelet counts and granule secretion can be associated with, but are not fully penetrant for ASD.

**Electronic supplementary material:**

The online version of this article (doi:10.1186/s13229-015-0051-y) contains supplementary material, which is available to authorized users.

## Background

Autism spectrum disorders (ASDs) are a heterogeneous group of neurodevelopmental disorders characterized by impairments in reciprocal social interactions, verbal and non-verbal communication, and stereotyped behavior. The prevalence of ASD is estimated to be around 1.5 % of the population and has risen rapidly the past decade making it now one of the most prevalent disorders in childhood [1]. Although ASD is a highly heritable disorder [2], the genetic cause is complex and involves a multitude of genes, genetic heterogeneity, imprinting as well as gene-environment interactions [3–5]. Gene pathway analysis studies have provided insight in the diverse nature of the genetic abnormalities, and it now becomes evident that the majority of genetic defects influence a limited number of core functional mechanisms in the brain [6–10]. These identified networks are mainly related to the neuronal synapse and include synaptic plasticity, structure, signaling, and transcriptional regulation. Studying synaptic function in vivo and modeling the effect of single gene mutations in vitro are challenging despite the availability of non-invasive technologies to study human brain function and the development of several animal models [11–13].

Blood platelets and neurons that seem to share some typical characteristics as subclinical platelet abnormalities were often detected in monogenetic and complex neurological diseases [14, 15]. Detailed functional platelet testing was performed to gain insights in the disease mechanisms of some monogenetic neuropathologies by elucidating abnormalities in transcription factors, membrane transporters, G-protein signal transduction, and cytoskeletal proteins (e.g., Rett syndrome [16], intellectual disability, and others [17–19]). However, in the early 1970s, studies focused mainly only on the overlapping serotonin metabolism in neurons and platelets [20, 21], both comprising the active transport system for serotonin [22], storage into specialized dense core granules, and availability of monoamine oxidase in mitochondria. Since then, additional research has shown that both neurons and platelets contain overlapping gene expression profiles, similar types of granules loaded with partially the same signaling molecules such as ATP and serotonin, and that both cell types use Ca^2+^, intracellular inositol phosphate and the prostaglandin pathway as initiators of granule secretion. Moreover, the highly specialized process of regulated granule trafficking and secretion found in neurons mirrors at least partially that of platelets [23, 24]. Abnormalities in the platelet serotonin metabolism have been described in different complex neurological diseases, including schizophrenia [25], migraine [26], and Alzheimer’s disease [27]. Elevated blood serotonin level is also the first quantitative trait found to be associated with ASD [28, 29]. A familial study showed that plasma free serotonin is correlated with ASD but is not different between ASD cases and their siblings or parents [30]. This study also pointed out that the difference in serotonin is due only to the serotonin of the platelet fraction as the association was not found for serotonin levels in platelet-poor plasma. A study by Piven J et al. showed that serotonin levels in platelet-rich plasma of ASD patients with affected siblings were significantly higher than probands without affected siblings, though, the latter cases still had serotonin levels significantly higher than controls [31]. A recent meta-analysis of all literature studies reporting ASD and control blood serotonin values concluded that elevated serotonin levels were recorded in 28.3 % in whole blood and 22.5 % in platelet-rich plasma samples of ASD patients, as reported in 15 and 4 studies, respectively, using different detection techniques [32]. It is difficult to make an overall conclusion concerning the true association between ASD and serotonin as potential biomarker since serotonin concentrations are highly dynamic in the central nervous system and around [33], and different methods to measure serotonin using different biomaterials (whole blood, platelets, platelet-rich, or platelet-poor plasma) do exist [34]. This, altogether, probably accounts for the differences amongst a large number of studies that report serotonin levels as being a possible true biomarker for ASD or not.

Platelets store and release upon activation of serotonin from their dense granules and therefore, we hypothesized that differences in the platelet serotonin metabolism can be due to abnormalities in platelet dense granule formation, transport, and secretion. Therefore, it is also important to study the morphology and release of the platelet dense granules in addition to measuring serotonin levels. Indeed, we previously observed an altered platelet dense granule morphology in three unrelated ASD patients with chromosomal translocations. It was shown that the identified ASD candidate genes *NBEA*, *SCAMP5*, and *AMISYN* involved in these translocations play a role in stimulated secretion of large dense-core vesicles [35, 36]. Interestingly, heterozygous NBEA-deficient mice also have autistic-like behavior problems [37] and a platelet dense granule morphology defect but these mice have normal serotonin levels in platelets and serum [38]. Therefore, we hypothesized that in at least a subgroup of ASD patients, a defect in synaptic neuronal signaling would be reflected by alterations in platelet dense granule secretion. Platelets offer a unique opportunity to assess the stimulated dense granule function, in a relatively noninvasive and high-throughput manner because they can be isolated from peripheral blood in a non-active state. We here evaluated platelet count, size, and activation in addition to the classic measurements of only plasma serotonin levels in a non-syndromic familial ASD cohort.

## Methods

### Study participants and clinical evaluations

Written informed consent was obtained for all studies from all participants and/or their legal representatives. The Medical Ethical Committee of the University Hospitals of Leuven approved this study (project number S50717). Participants from the larger ASD family cohort recruited by the Leuven Autism Research (LAuRes) consortium that were previously described [39] were asked to participate additionally in this platelet study. A total (*n* = 448) of 159 ASD patients and 289 first-degree relatives (103 unaffected siblings and 186 parents) from the LAuRes cohort agreed to enroll in this study. Further characteristics of the ASD family and unrelated healthy adult and pediatric control cohorts are presented in Table 1. Briefly, the diagnosis ASD was made in children by multidisciplinary teams in a standardized way according to DSM-IV-TR criteria [40]. Parents were asked to fill out the developmental, dimensional and diagnostic interview (3di) [41] and social responsiveness scale (SRS) [42] about their children under 18 years old. We also asked them to have an adult SRS filled out about themselves by their partner. We included only children under 18 years of age and excluded half-siblings. Besides obtaining the adult SRS scores, parents were not additionally screened for ASD but five fathers were previously diagnosed with ASD and were excluded from the analysis of this study. Individuals who used platelet function-interfering medication preceding blood drawing were also excluded from the study (Additional file 1: Table S1). The adult control cohort for the platelet functional tests included 45 unrelated adults from the general population that specifically consented for functional platelet testing. Since the platelet counts and mean platelet volume (MPV) are age dependent, we also included data from a pediatric control cohort (*n* = 65) that was previously recruited for a different study (obesity medication, blood sampling before onset of the study) to compare these parameters. Written informed consent from these participants was given for the use of platelet count and size in research context. The ATP secretion and aggregation studies were not included in the pediatric study and therefor not available. Unfortunately, these adult and pediatric control cohorts were only assumed to be non-ASD cases but they were not specifically tested using the DSM-IV-TR or SRS systems. We recorded no data related to cardiovascular risk factors (smoking, hypertension, weight, etc.) for any of the participants.

**Table 1:** Demographics of the study participants

	ASD family cohorts			Control cohorts	
	ASD	Siblings	Parents	Pediatric	Adult
*N*	159	103	186	65	45
Gender (male-female)	125–34	34–69	87–99	31–34	22–23
Age (years)	11.9 ± 3.8	13.0 ± 4.7	43.0 ± 4.4	15.6 ± 3.9	36.5 ± 10.9
Intellectual disability (%)	18 (11)	1 (1)	0 (0)	0 (0)	0 (0)
SRS score	89.6 ± 16.5 (3)	50.0 ± 9.7 (3)	48.0 ± 8.7 (15)	nd	nd

Data are expressed as mean ± standard deviation (missing values) or *n* (%)

*ID* intellectual disability is defined as IQ < 70, *SRS* social responsive scale, *nd* not determined

### Platelet count, size, and functional platelet studies

Blood (40 mL) from each individual was anti-coagulated with 129 mmol/L trisodium citrate (9:1) and used within 2 h to obtain platelet-rich plasma (PRP). Hemolytic or lipemic samples were excluded for further studies (Additional file 1: Table S1). Platelet count in the PRP was adjusted to 250 × 10^9^ platelets/L. The PRP was used for functional platelet studies as described [17, 18, 43]. Briefly, aggregation studies were performed on two dual-channel Chrono-Log aggregometers (Chronolog, Havertown, PA, USA) by adding 1.25 μg/mL epinephrine, 2.5 μg/mL epinephrine or 1 mM arachidonic acid (AA; Sigma Chemical Co, St Louis, MO). The maximal aggregation response (amplitude, %) and the rate of aggregation (slope) are automatically measured and reported by the aggregometer. Platelet secretion was determined by measuring the release of adenosine-triphosphate (ATP) using luciferin/luciferase reagent (Kordia, Leiden, the Netherlands) after stimulation with 10 μM adenosine-diphosphate (ADP; Sigma Chemical) or 1 mg/mL Horm collagen (Nycomed Arzenmittel, Munich, Germany). Though all participants were questioned in detail for medication use to exclude potential drug-interfering effects on platelet activation, the AA-induced aggregation response was performed to exclude potential side effects of aspirin or other nonsteroidal anti-inflammatory drugs (NSAIDs)-like drugs that typically interfere with AA responses.

Whole blood cell counts were obtained with an automated cell counter (Cell-Dyn 1300; Abbott Laboratories, Abott Park, IL, USA) using EDTA-treated blood samples for all individuals except for the adult controls for which no EDTA-treated samples were collected. Serotonin was measured in platelet-poor plasma (from EDTA-treated blood samples) in a smaller group of age and gender-matched ASD children (*n* = 30, age *M* = 14 range 9–18), non-ASD siblings (*n* = 30, age *M* = 14 range 9–18), and pediatric controls from whom samples were available (*n* = 16, age *M* = 14 range 9–20) using a competitive ELISA according to the manufacturers instructions (IBL International, Hamburg, Germany). C-reactive protein (CRP) levels were measured in citrated plasma samples from the same subjects as mentioned for the serotonin measurements using the human (CRP) Quantikine ELISA kit according to the instructions (R & D Systems inc, Minneapolis, MN).

### Statistical analysis

Spearman correlations, Fisher’s exact, and Mann-Whitney *U* tests were used for exploratory purposes. For each of the responses (platelet count, mean platelet volume, aggregations, and ATP secretions) separately, a linear mixed model was used to compare the level between children with ASD, siblings, parents, and either children or adult controls. The included random effects in the mixed models imposed restrictions on the covariance matrix of the responses predicated by genetic theory. More specifically, they refer to an additive genetic component and a common environmental component [44] to account for the fact that ASD children and siblings are raised in the same family setting. Gender was added as covariate in the mixed model analysis. In the correlation analysis, children with ASD, siblings, and parents are compared with gender and the SRS score as covariates. Tukey’s adjustments for multiple testing are used for the pairwise comparisons between groups in all analyses. The residual variability of the responses is allowed to be group-specific. *p* values smaller than 0.05 are considered significant. All analyses have been performed using SAS software, version 9.2 of the SAS System for Windows.

## Results

### Platelet count and size in ASD family and pediatric control cohorts

**Fig. 1:**
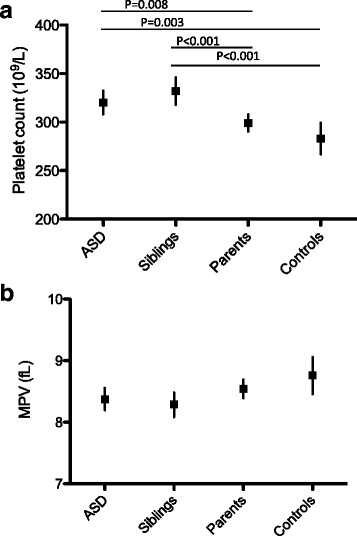
Platelet count and size in ASD patients, siblings, parents, and pediatric controls. Graphs indicating gender-corrected least squares means and 95 % CI values based on the linear mixed model analysis of platelet count (**a**) and platelet size (**b**) in 159 ASD patients, 103 siblings, 186 parents, and 65 pediatric controls measured in whole blood. Tukey-Kramer-adjusted *p* values of the pairwise comparisons in four groups are indicated when they are significant (*p* < 0.05)

Platelet count and size as represented by the mean platelet volume (MPV) were assessed in individuals with ASD, their first-degree relatives and pediatric controls (Table 1 for cohort characteristics). ASD patients and their siblings have higher platelet counts compared to age-matched pediatric controls (Table 2). Within the ASD family subgroups, total platelet count did not differ between genders, except for the parent sample where the mean platelet count was higher for mothers (*M* = 308, SD 6.4) compared to fathers (*M* = 290, SD 6.1, *p* = 0.04). A linear mixed model showed a significant effect of group on platelet count (*p* < 0.001). Post-hoc pairwise tests revealed significant differences between ASD patients and non-ASD siblings compared to parents and pediatric controls, but not between ASD patients and their unaffected siblings (Fig. 1a). Furthermore, we observed mild thrombocytosis (platelet counts above the normal upper limit of 450 × 10^9^/L blood) in several ASD patients, siblings, and parents (Table 2).

**Table 2:** Platelet count and size in ASD family cohorts and pediatric controls

	ASD	Siblings	Parents	Pediatric controls
*N*	158^a^	103	186	65
Platelet count (×10^9^/L blood)	318.7 ± 71.9	337.2 ± 67.6	300.6 ± 62.0	283.4 ± 67.2
Thrombocytosis	6 (3.8)	4 (4.0)	4 (2.1)	0 (0)
MPV (fL)	8.2 ± 1.0 (1)	8.3 ± 1.1	8.5 ± 1.1	8.8 ± 1.2

Data are expressed as mean ± standard deviation (missing values) or *n* (%). Values are represented as unadjusted raw values. Thrombocytosis is defined by a platelet count >450 × 10^9^/L. Platelet size is indicated with mean platelet volume (MPV)

^a^An EDTA blood sample was missing from one ASD patient from which platelet count and MPV could therefore not be measured. A MPV measurement for a second ASD patient was missing due to sampling failure of the counter

Whole blood measurements in normal human subjects always reveal an inverse relation between platelet count and MPV [45, 46]. This inverse relationship was also found for ASD patients (*N* = 157, rho = −0.48, *r*^2^ = 0.23, *p* < 0.001). As therefore expected, the MPV was decreased for both ASD patients and siblings compared to the pediatric control group (Table 2). However, linear mixed model analysis showed no significant differences in MPV amongst the groups (Fig. 1b).

### Platelet activation studies in ASD family and adult control cohorts

Agonist-induced platelet activation was assessed in ASD family and adult control cohorts using the in vitro aggregation test. Platelet activation studies with arachidonic acid (AA) being the precursor of thromboxane A2 (TxA2), a strong stimulator of platelet activation, were mainly included to control for medication-interfering drugs (such as NSAIDs) that typically affects the AA-induced platelet aggregation (AA-induced platelet activation pathway shown in red in Additional file 2: Figure S1). No significant difference in the maximal AA-induced aggregation amplitude was observed between the different ASD family subgroups or the control cohort (Additional file 2: Figure S2).

**Fig. 2:**
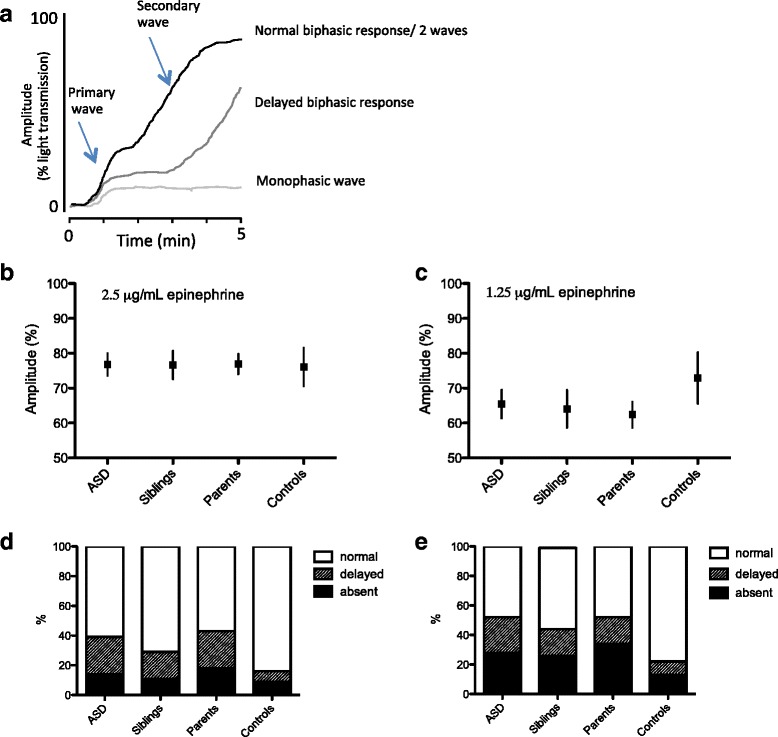
Aggregation responses following platelet activation with epinephrine in ASD patients, siblings, parents, and adult controls. **a** Schematic representation of platelet aggregation curve induced by 1.25 μg/mL epinephrine showing monophasic (*1 wave*), biphasic (*2 waves*), or delayed biphasic aggregation curves. Graphs indicating gender-corrected least squares means and 95 % CI values based on the linear mixed model analysis of maximal aggregation amplitude in response to **b** 2.5 μg/mL epinephrine and **c** 1.25 μg/mL epinephrine measured in 159 ASD patients, 103 siblings, 186 parents, and 45 adult controls measured in platelet-rich plasma. Tukey-Kramer-adjusted *p* values of the pairwise comparisons in four groups are indicated when they are significant (*p* < 0.05). Graphs indicating percentage of individuals per group with either normal, delayed, or absent secondary wave aggregation following **d** 2.5 μg/mL epinephrine, or **e** 1.25 μg/mL epinephrine

We next assessed the platelet activation response to the granule secretion-dependent platelet activating agonist epinephrine. Epinephrine is a weak agonist that induces the release of intracellular calcium leading to a “primary wave” of aggregation. This initial aggregation response can be followed by a “secondary wave” if TxA2, ADP, and other stimulatory molecules are released from the platelet granules to amplify the primary aggregation response (illustrated in Fig. 2a and pathway shown in blue in Additional file 2: Figure S1). The maximal amplitude for the epinephrine-induced platelet aggregation is therefore determined by both waves. Using the linear mixed model analysis, no significant different in maximal amplitude was measured in stimulated platelets from ASD patients, non-ASD siblings, parents, and adult controls using either intermediate (2.5 μg/mL) or low (1.25 μg/mL) concentrations of epinephrine (Fig. 2b, c). Though not significant, a trend for lower maximal aggregation amplitudes was observed after stimulation with 1.25 μg/mL epinephrine in all ASD family subgroups when compared with the adult control cohort. Since the maximal amplitude is a combination between primary and secondary aggregation responses, we also directly scored the secretion-dependent secondary wave as being present, delayed, or absent (as illustrated in Fig. 2a). Interestingly, for the adult control cohort, only 16 % of all participants showed a delayed or absent secondary wave towards 2.5 μg/mL epinephrine stimulation compared to 39, 30, and 42 % in the ASD, non-ASD sibling, and parent groups, respectively (*p* = 0.002) (Fig. 2d). This secondary wave scoring difference was even more pronounced for stimulations with the low dose of epinephrine (ASD 52 %, siblings 45 %, parents 53 %, controls 22 %, *p* = 0.002 (Fig. 2e)). However, no scoring differences were observed between ASD and non-ASD participants within the family cohort.

### Platelet dense granule ATP secretion in ASD family and adult control cohorts

**Fig. 3:**
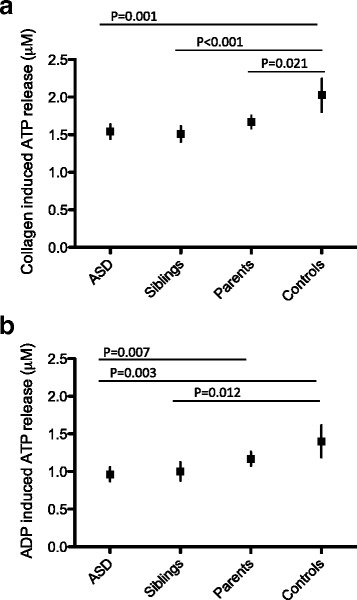
ATP secretion response following platelet activation in ASD patients, siblings, parents, and adult controls. Graphs indicating gender-corrected least squares means and 95 % CI values based on the linear mixed model analysis of ATP release in response to **a** collagen and **b** ADP in 159 ASD patients, 103 siblings, 186 parents, and 45 adult controls measured in platelet-rich plasma. Tukey-Kramer-adjusted *p* values of the pairwise comparisons in four groups are indicated when they are significant (*p* < 0.05)

As ASD family participants have a delayed or absent granule secretion-dependent secondary wave after stimulation with epinephrine, abnormal platelet dense granule secretion would further support this finding. Platelet stimulation results in the fusion of granules with plasma membranes to release their content [47]. We measured the release of ATP stored in dense granules after platelet stimulation with the different agonist collagen and ADP in platelet-rich plasma from ASD family and control cohort participants (secretion pathway induced by ADP and collagen is illustrated in Additional file 2: Figure S1). Collagen is a strong agonist that induces platelet aggregation and TxA2 synthesis with simultaneous platelet granule secretion. Using a dose of 2 μg/mL collagen, we found that ATP release was significantly lower in the ASD, non-ASD sibling, and parent groups compared to controls but also amongst the ASD and sibling group vs. the parents, as determined in the linear mixed model analysis (Fig. 3a). ADP is a weak agonist that is also stored in the dense granules of platelets. Full granule secretion after ADP stimulation can only occur in response to a positive feedback loop following platelet aggregation and TxA2 synthesis. Following activation with 10 μM ADP, ATP release was significantly lower in the ASD patients and siblings but not in parents compared to controls using linear mixed model analysis (Fig. 3b). Significant differences were also observed between ASD patients compared to parents, but not between ASD patients and their non-ASD siblings.

### Plasma serotonin levels in ASD family and pediatric control cohorts

**Fig. 4:**
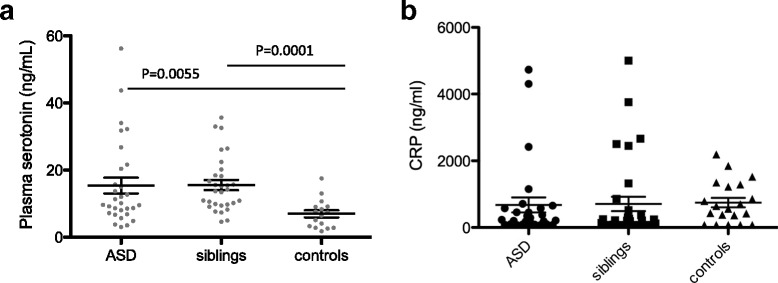
Plasma serotonin and CRP levels in ASD patients, siblings, and pediatric controls. **a** Plasma serotonin levels were measured using an ELISA in a selection of ASD patients (*n* = 30), siblings (*n* = 30), and age-matched controls (*n* = 16). *Line* and *whiskers* indicate mean and SEM. Groups were compared using the Mann-Whitney *U* test. **b** Plasma CRP levels were measured using an ELISA in the same selection of ASD patients (*n* = 30), siblings (*n* = 30), and age-matched controls (*n* = 16). *Line* and *whiskers* indicate mean and SEM. Groups were compared using the Mann-Whitney *U* test

Regulation of plasma serotonin levels involves the activity and presence of the serotonin transporter at the plasma membrane of platelets. In addition, platelets express the G protein-coupled serotonin receptor that by itself cannot trigger platelet aggregation but can participate with other platelet agonists to obtain full platelet aggregation. High serotonin levels in plasma can therefore result in a higher sensitivity towards weak agonists such as ADP and epinephrine. We measured serotonin levels in randomly selected subgroup of ASD patients and siblings and found elevated plasma serotonin compared to controls (Fig. 4a). Again, no difference was observed between ASD and non-ASD siblings. Therefore, the increased sensitivity of control platelets to epinephrine or ADP stimulation cannot be attributed to higher serotonin plasma levels. In addition, as it is known that platelet activity is sensitive to the inflammation status of the blood [48], we also determined C-reactive protein (CRP) levels in the plasma samples from the same group of patients, siblings, and controls but found no significant differences between the groups (Fig. 4b).

### Clinical correlates of platelet function and counts

Given the association between platelet count and function changes and the presence of ASD in the family, we explored the possibility that these parameters could correlate with autism traits within the families. We therefore evaluated the association of platelet counts, aggregation, ATP secretion, and serotonin levels with SRS scores of ASD patients, siblings, and parents. Interestingly, we found small but significant correlations between SRS scores and mean platelet volume (*r* = −0.11, *p* = 0.02), aggregation with 2.5 μg/mL epinephrine (*r* = −0.12, *p* = 0.01), ATP release with collagen (*r* = −0.11, *p* = 0.02), and ATP release with ADP (*r* = −0.13, *p* = 0.01). These correlations lost significance when assessed for the individual groups within the ASD family cohort probably because of the small sample sizes.

## Discussion

Platelet function studies in non-syndromic ASD families revealed abnormalities in platelet count, epinephrine-induced secondary wave aggregation responses, and dense granule ATP secretion not only in ASD patients but also in their first-degree relatives, namely their parents and non-ASD siblings. A weakness of our study is that we have only used adult controls for functional platelet studies and pediatric controls for platelet count and volume measurements. In addition, we could not test these control cohorts for ASD features. All analyses were controlled for a gender effect. We analyzed the effect of age on our studied parameters and found a significant effect within the children group for platelet counts only. This might be caused by an effect of puberty, as the number of post-puberty in the ASD group is lower than that in the siblings (15 % for ASD vs 23 % for siblings). However, the small group size made it impossible to distinguish between these age groups by lack of statistical power.

The observed platelet functional abnormalities in ASD cases and their non-affected siblings are typically related to a defect in dense granules as we have found before in some ASD patients with chromosomal abnormalities [35]. This type of platelet defect is mild and is not expected to result in obvious clinical bleeding problems. The functional platelet test as performed in the current study cannot be used for diagnosis or even for prognosis of ASD as this type of defect is not specific for ASD. Mild dense granule secretion defects were previously also found for patients with a monogenetic neuropathology [16, 17]. However, we believe that the study is important as a subgroup of ASD patients and first-degree relatives are identified with an obvious platelet dense granule secretion defect, and this population will further be investigated by more specific platelet tests that are not feasible for large scale studies such as morphological dense granule investigations by electron microscopy and dense granule formation, transport, and secretion assays using specific markers in platelets and during megakaryopoiesis [49]. In addition, ASD patients with platelet abnormalities will be selected for further next generation sequencing studies since important platelet-based tools are available that will assist candidate gene selection. We expect that the causative gene is expressed by megakaryocytes and detailed gene expression data are now available for all blood cells [50]. An important set of next generation sequencing data is available for 707 patients with bleeding and platelet disorders (BPD) that will be used as internal control to filter for candidate gene variants [51]. ASD patients with platelet defects identified in the current study will now be sequenced and analyzed in parallel with this large dataset of BPD. Interestingly, the initial analysis of these 707 BPD patients that did not yet include samples from this familial ASD study showed a significant association between human phenotype ontology terms for the “blood” and “brain” systems.

We realize that the secretion-dependent aggregation and ATP dense granule secretion abnormalities in children with ASD, their siblings and their parents can be caused by many different genes. There is a plethora of genes that could cause abnormalities in platelet function, many of which have been described in the field of bleeding disorders [52]. An altered platelet function without associated bleeding problems has also been described in a number of monogenetic and complex neurological disorders [14]. These alterations include changes in MAO activity, serotonin metabolism, platelet aggregation, and dense granule secretion in schizophrenia [25], platelet morphology and MAO metabolism in Parkinson’s disease [53, 54], and altered platelet serotonin metabolism in depression [55]. Recently, platelet defects were also described in idiopathic ASD patients carrying de novo chromosomal translocations near NBEA, SCAMP5, and AMYSIN, and in vitro gene silencing studies linked these genes to a role in vesicle secretion [35]. These important findings underscore the possibility that single genetic defects can cause both a neurological phenotype and a platelet secretion defect.

Platelet counts are kept at constant levels by an elegant feedback mechanism provided by the cytokine thrombopoietin (TPO), which is produced in liver and kidneys. Increased levels of TPO induce production of platelets by megakaryocytes in the bone marrow, and platelets themselves serve as a clearance sink for TPO keeping production in balance [56]. To our knowledge, altered plasma TPO levels have not been documented in ASD. However, a plausible explanation for the higher platelet counts might also simply be deduced from our observation that platelets of ASD patients and family members are less prone to activation during blood drawing. Their circulating platelets also have reduced tendency to adhere in vivo to endothelial cells and neutrophils. The increase in platelet counts in ASD might therefore simply reflect their relative resting state under basal conditions. We found no significant change in MPV as previously reported by others [57].

Consistent with previous reports, serotonin levels were increased in this cohort of patients with ASD and their siblings [32]. Physiologically, elevated blood serotonin levels have been linked to the density of the serotonin transporter at the plasma membrane of platelets [58]. The regulation of its activity and trafficking involves a complex of proteins including the αIIbβ3 integrin receptor that is responsible for sustained platelet activation. Reduced function of this receptor was recently linked to reduced uptake of serotonin by the serotonin transporter [59]. Since we find reduced platelet aggregation and secretion in ASD, we hypothesize that the increased serotonin levels are caused by reduced uptake rather than by increased release from platelets.

Using the linear mixed model that corrects for a common genetic and environmental background [44], we could not detect any significant platelet count or function differences between ASD patients and their non-ASD siblings. This finding is in accordance with the idea that development of ASD may involve the interaction between rare genetic variants with moderate effects in different genes rather than the addition of Mendelian inheritance. ASD is a complex polygenetic disease for which, a cumulative effect of low-penetrance genetic factors or gene-dosage effect is expected that would have partial effects on the platelets of non-ASD siblings and parents. Further platelet studies in larger cohorts are needed to study such cumulative effects. Another possible weakness of this study is that the platelet function tests in ASD children were compared with adult controls only. However, platelet function is to our knowledge not age dependent (unlike platelet count and MPV) but additional independent studies are needed to confirm our initial findings.

## Conclusions

Though this study was limited by potential confounders such as cohorts biased and the missing information related to comorbidities and medications, we have found evidence for increased platelet counts, and decreased platelet secretion-dependent aggregation, and ATP dense granule secretion responses in ASD patients and first-degree relatives. Specific testing for platelet dense granule secretion in larger ASD cohorts must be performed to find out if this test can be used as a detection method for ASD.

## Availability of supporting data

The data sets supporting the results of this article are included within the article and its additional files.

## Additional files

Additional file 1: Table S1. Criteria leading to exclusion of individuals in the platelet study. (DOC 33 kb) Additional file 2: Figure S1. Platelet activation pathways investigated in this study. Platelet aggregation with the strong agonist arachidonic acid (AA) stimulates platelets via the formation of thromboxane A2 (TxA2) by the enzyme COX1 to finally active the G protein-coupled thromboxane receptor that will result in calcium efflux and the release of alpha (α) and dense (δ) granules. A high dose of AA does not require platelet secretion for full platelet activation. Activation with the weak agonist epinephrine (EPI) will partially stimulate platelets via the adrenergic receptor but will require platelet δ granule secretion (with the release of ADP and TxA2) to obtain full platelet activation. Platelet ATP secretion from δ granules was measured after platelet activation with collagen and ADP that stimulate different pathways to result in the release of ATP from dense granules that was measured using a lumino-aggregometer. Serotonin was measured in plasma after isolation of platelets by centrifugation. It is known that serotonin can active it G protein-coupled receptor but it is a weak agonist that is not able to induce platelet aggregation without stimulation with another agonist. Normal plasma concentrations of serotonin are unable to result in full platelet aggregation. Additional file 2: Figure S2 Platelet aggregation with arachidonic acid. Platelet aggregation in response to 1 mM AA in 159 ASD patients, 103 siblings, 186 parents, and 41 adult controls measured in platelet-rich plasma. Graphs indicating means and 95 % CI value. (PDF 97 kb)

## Abbreviations

AA: arachidonic acid
AD: Alzheimer’s disease
ADP: adenosine diphosphate
ASD: autism spectrum disorder
ATP: adenosine triphosphate
MAO: monoamine oxidase
MPV: mean platelet volume
NSAID: nonsteroidal anti-inflammatory drug
PRP: platelet rich plasma
SRS: social responsiveness score
TPO: thrombopoietin
TxA2: thromboxane A2
