# Supplementary material for: Platelet studies in autism spectrum disorder patients and first-degree relatives
Source: Mol Autism. 2015 Oct 23;6:57. doi: 10.1186/s13229-015-0051-y (PMC4619313; doi:10.1186/s13229-015-0051-y)
Supplement: Additional file 1: Table S1. — Criteria leading to exclusion of individuals in the platelet study. (DOC 33 kb) [file 13229_2015_51_MOESM1_ESM.doc]

Supplemental Table 1: Criteria leading to exclusion of individuals in the platelet study

|  |  | ASD | | Siblings | | Parents | | Total |
| --- | --- | --- | --- | --- | --- | --- | --- | --- |
|  |  | M | F | M | F | M | F |  |
| medication | Risperidon | 17 | 3 | 1 | 1 |  |  | 22 |
|  | Anti-convulsant | 2 | 2 |  |  | 1 | 1 | 6 |
|  | NSAID | 2 | 1 | 2 | 2 | 11 | 15 | 33 |
|  | Acetaminophen | 3 |  | 1 | 1 | 2 | 4 | 11 |
|  | Other meds | 1 |  | 1 |  | 4 | 1 | 7 |
| Insufficient quality of sample | | 1 |  | 1 |  | 3 | 5 | 10 |
| Diagnosis of ASD in adults | |  |  |  |  | 5 |  | 5 |
| Total excluded | |  |  |  |  |  |  | 94 |
