# Supplementary figures and images for: Platelet studies in autism spectrum disorder patients and first-degree relatives
Source: Mol Autism. 2015 Oct 23;6:57. doi: 10.1186/s13229-015-0051-y (PMC4619313; doi:10.1186/s13229-015-0051-y)

Supplemental Figure 1

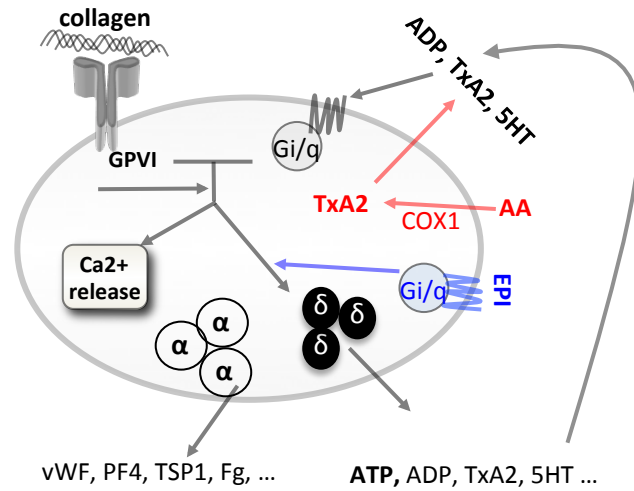

Supplemental Figure 2

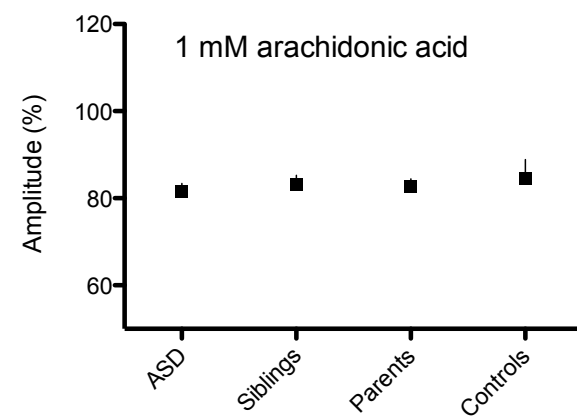

Supplement: Additional file 2: Figure S1. — Platelet activation pathways investigated in this study. Platelet aggregation with the strong agonist arachidonic acid (AA) stimulates platelets via the formation of thromboxane A2 (TxA2) by the enzyme COX1 to finally active the G protein-coupled thromboxane receptor that will result in calcium efflux and the release of alpha (α) and dense (δ) granules. A high dose of AA does not require platelet secretion for full platelet activation. Activation with the weak agonist epinephrine (EPI) will partially stimulate platelets via the adrenergic receptor but will require platelet δ granule secretion (with the release of ADP and TxA2) to obtain full platelet activation. Platelet ATP secretion from δ granules was measured after platelet activation with collagen and ADP that stimulate different pathways to result in the release of ATP from dense granules that was measured using a lumino-aggregometer. Serotonin was measured in plasma after isolation of platelets by centrifugation. It is known that serotonin can active it G protein-coupled receptor but it is a weak agonist that is not able to induce platelet aggregation without stimulation with another agonist. Normal plasma concentrations of serotonin are unable to result in full platelet aggregation. Additional file 2: Figure S2 Platelet aggregation with arachidonic acid. Platelet aggregation in response to 1 mM AA in 159 ASD patients, 103 siblings, 186 parents, and 41 adult controls measured in platelet-rich plasma. Graphs indicating means and 95 % CI value. (PDF 97 kb) [file 13229_2015_51_MOESM2_ESM.pdf]
